# A Genome-Wide Analysis of Small Regulatory RNAs in the Human Pathogen Group A *Streptococcus*

**DOI:** 10.1371/journal.pone.0007668

**Published:** 2009-11-02

**Authors:** Nataly Perez, Jeanette Treviño, Zhuyun Liu, Siu Chun Michael Ho, Paul Babitzke, Paul Sumby

**Affiliations:** 1 Center for Molecular and Translational Human Infectious Diseases Research, The Methodist Hospital Research Institute, Houston, Texas, United States of America; 2 Department of Biochemistry and Molecular Biology, The Pennsylvania State University, University Park, Pennsylvania, United States of America; Cairo University, Egypt

## Abstract

The coordinated regulation of gene expression is essential for pathogens to infect and cause disease. A recently appreciated mechanism of regulation is that afforded by small regulatory RNA (sRNA) molecules. Here, we set out to assess the prevalence of sRNAs in the human bacterial pathogen group A *Streptococcus* (GAS). Genome-wide identification of candidate GAS sRNAs was performed through a tiling Affymetrix microarray approach and identified 40 candidate sRNAs within the M1T1 GAS strain MGAS2221. Together with a previous bioinformatic approach this brings the number of novel candidate sRNAs in GAS to 75, a number that approximates the number of GAS transcription factors. Transcripts were confirmed by Northern blot analysis for 16 of 32 candidate sRNAs tested, and the abundance of several of these sRNAs were shown to be temporally regulated. Six sRNAs were selected for further study and the promoter, transcriptional start site, and Rho-independent terminator identified for each. Significant variation was observed between the six sRNAs with respect to their stability during growth, and with respect to their inter- and/or intra-serotype-specific levels of abundance. To start to assess the contribution of sRNAs to gene regulation in M1T1 GAS we deleted the previously described sRNA PEL from four clinical isolates. Data from genome-wide expression microarray, quantitative RT-PCR, and Western blot analyses are consistent with PEL having no regulatory function in M1T1 GAS. The finding that candidate sRNA molecules are prevalent throughout the GAS genome provides significant impetus to the study of this fundamental gene-regulatory mechanism in an important human pathogen.

## Introduction

Small RNA molecules with regulatory activities have been described in all three domains of life, indicative of an ancient evolutionary history. In prokaryotes, small RNAs with regulatory functions include riboswitches [1], transfer-messenger RNA (tmRNA) [2], 4.5S RNA [3], 6S RNA [4], and small regulatory RNAs (sRNAs) [5]. sRNAs are key mediators of virulence gene expression in some pathogens, and can regulate diverse cellular processes such as the stress and adaptive responses [6], [7]. The majority of described sRNAs regulate through a mechanism involving complementary base-pairing with the 5′ end of target mRNAs, blocking access to the ribosome binding site and/or start codon. In addition to blocking mRNA translation, sRNA:mRNA duplex formation can target both RNA molecules for degradation by double-stranded RNA cleaving ribonucleases (e.g. RNase III) [8]. The post-transcriptional regulation afforded by sRNAs means they impose a regulatory step independent of, and epistatic to, target mRNA transcriptional signals [5].

The bacterial pathogen group A *Streptococcus* (GAS; *Streptococcus pyogenes*) is the etiological agent of several human diseases, including pharyngitis, impetigo, acute rheumatic fever, streptococcal toxic-shock-like syndrome, and necrotizing fasciitis [9]. The ability of GAS to cause such a wide variety of human infections is at least in part due to its ability to coordinately regulate gene expression to microenvironment specific conditions [10], [11]. GAS transcription is regulated through the concerted action of 13 conserved ‘two-component’ signal transduction systems (named due to the functional linkage of two independent proteins, a sensor kinase and a response regulator) and >60 ‘stand-alone’ transcription factors (named due to their ability to independently regulate transcription) [10], [12].

To date only three sRNAs have been described in GAS, the pleiotropic effect locus (PEL) [13], [14], the fibronectin/fibrinogen binding/hemolytic activity/streptokinase regulator X (FASX) [15], and the RofA-like protein IV regulator X (RIVX) [16]. PEL, FASX, and RIVX are all reported to regulate GAS virulence factor expression, providing for the possibility that sRNAs represent a major mechanism of virulence-regulation in this pathogen. To start to address this issue we determined the prevalence, location, orientation, and temporal transcription pattern of candidate GAS sRNAs. The mapping and initial characterization of sRNAs throughout the GAS genome provides significant impetus to the study of these molecules as potential regulators of virulence in GAS and related pathogens.

## Materials and Methods

### Bacterial strains and culture conditions

Strain MGAS2221 is representative of the highly virulent M1T1 GAS clone responsible for significant morbidity and mortality since the mid-1980s in the U.S., Canada, and Western Europe [17], [18]. Strain information for the nine serotype M1 isolates used in this study is listed in Table S5. GAS strains were grown *in vitro* in Todd-Hewitt broth with 0.2% yeast extract (THY broth) at 37°C (5% CO_2_).

### Total RNA isolation

Frozen GAS cell pellets were resuspended in 100 µl TE buffer and transferred to 2 ml tubes containing fine glass shards (lysing matrix B tubes, MP Biomedicals). Tubes were placed into a glass bead beater (FastPrep machine, THERMO 101) and processed for 15 s at speed 4. Tubes were centrifuged for 5 s at 14,000 *g* to reduce foaming and an additional processing in the FastPrep machine was performed following addition of 650 µl of buffer RLT (Qiagen Inc.). Samples were centrifuged for 30 s at 14,000 *g* to collect contents and 600 µl transferred to a 1.5 ml tube containing 900 µl 100% ethanol. RNA samples were subsequently bound to, washed on, and eluted from, RNeasy columns (Qiagen Inc.) as per the manufacturers' miRNeasy protocol. Contaminating genomic DNA was removed from eluted RNA samples via four 30 min incubations at 37°C with 2 µl TURBO DNase-free (Applied Biosystems), with DNA removal being verified by PCR.

### Microarray identification of GAS sRNAs

A custom-made microarray (Affymetrix Inc.) was used to identify GAS sRNAs. The microarray consisted of overlapping 25mer oligonucleotides tiled on both strands of intergenic regions within the MGAS2221 genome. On average there were 17 nucleotides of overlap between adjacent probes. For each perfect match (PM) probe a corresponding mismatch (MM) probe was included on the array. MM probes are identical in sequence to PM probes with the exception that the central base of each 25mer probe is substituted. Subtracting MM probe hybridization signal intensity from that of the PM probe reduces background noise, increasing sensitivity.

Triplicate cultures of GAS strain MGAS2221 were grown at 37°C (5% CO_2_) in THY broth to the mid-exponential (O.D._600_ ∼0.5) phase of growth. Recovered GAS were incubated at room temperature for 5 min following addition of 2 volumes of RNAprotect (Qiagen Inc.) to maintain RNA integrity. GAS were harvested by centrifugation, quick frozen in liquid nitrogen, and stored at −80°C. Total RNA was isolated as described above. GAS RNA samples were quantified using the 2100 BioAnalyzer system (Agilent Technologies) and converted to cDNA using reverse transcriptase (Superscript III, Invitrogen Corp.) with random hexamers as per the manufacturers' protocol. Following cDNA synthesis, RNA was removed via NaOH hydrolysis and the cDNA quantified, again using the 2100 BioAnalyzer. Identical concentrations of individual cDNA samples were fragmented with DNase I to an average size of ∼50 bases before biotin labeling using terminal transferase (Promega) and the Affymetrix labeling kit. Labeled cDNAs were hybridized to the custom microarray at 42°C for 16 h. Arrays were processed (washed, stained, scanned) as per the Affymetrix protocol for low GC% bacteria (protocol FS450_0005). GeneChip Operating Software v1.4 (GCOS, Affymetrix Inc.), Tiling Analysis Software (TAS, Affymetrix Inc.), and Integrated Genome Browser software (IGB, Affymetrix Inc.) were used to generate probe specific signal intensities, normalize samples, generate *P*-values (via Wilcoxon signed rank test), and enable visualization of signal/*P*-value data in context of genome location. All data is MIAME compliant and the raw data has been deposited at the MIAME compliant Gene Expression Omnibus (GEO) database at National Center for Biotechnology Information (http://www.ncbi.nlm.nih.gov/geo) and are accessible through accession number GSE17790.

### Northern blot analysis

Total RNA was isolated from strain MGAS2221 during early exponential (O.D._600_ ∼0.2), late exponential (O.D._600_ ∼0.8), early stationary (O.D._600_ ∼1.2), and late stationary (O.D._600_ ∼1.7) phases of growth as described[19]. RNA samples (6 µg per growth phase) were loaded onto a 5% TBE-Urea gel and separated by electrophoresis. Biotinylated RNA size standards ranging in size from 100 nucleotides to 1,000 nucleotides (Biotinylated RNA century-plus marker, Applied Biosystems) were used to enable size determination of detected transcripts. RNA was transferred to nylon membrane via electroblotting, UV cross-linked, and probed overnight with an *in vitro* transcribed probe complementary to a candidate sRNA. *In vitro* transcribed probes were generated using the Strip-EZ T7 kit (Applied Biosystems), enabling membranes to be stripped and re-probed multiple times. DNA templates for *in vitro* transcription reactions were generated by PCR, with one primer containing the T7 promoter sequence (Table S2). On average probes were 80 nucleotides in length but ranged from 70 to 300 nucleotides. RNA probes were labeled with biotin prior to hybridization (Brightstar psoralen-biotin labeling kit, Applied Biosystems). Following washes Northern blots were developed (Brightstar biodetect kit, Applied Biosystems) and exposed to autoradiography film.

For Northern blots comparing sRNA expression between representative strains of 8 GAS serotypes total RNA was isolated during exponential (O.D._600_ ∼0.4) and early stationary (O.D._600_ ∼1.2) phases of growth in THY broth. For Northern blots comparing sRNA expression between 9 representative serotype M1 strains total RNA was isolated only during the exponential phase. Northern blots were created and processed as described above only using 4 µg RNA for exponential phase cultures and 6 µg RNA for early stationary phase cultures.

### 5′ RACE to determine sRNA transcriptional start sites

The 5′ rapid amplification of cDNA ends (RACE) system (Invitrogen) was used as per the manufacturer's instructions. Briefly, sRNA-specific primers (GSP1 primers) were used to prime the reverse transcription of RNA from strain MGAS2221 (Table S2). Synthesized cDNA was purified and a poly(C) 3′ tail added using terminal transferase. Tailed cDNAs were used as template in a PCR with downstream primer GSP2 (downstream relative to primer GSP1) and a primer that ended with a poly(G) sequence (primer AAP; Invitrogen). AAP primer specificity was assayed through use of control PCRs using untailed cDNA as template. Products were visualized on standard 2% agarose gels stained with ethidium bromide. PCR-generated bands were gel extracted, cloned (pCRII-TOPO; Invitrogen), and sequenced.

### Measurement of sRNA stability

To gain insight into the stability of candidate sRNAs we inhibited RNA synthesis in exponential (O.D. ∼0.4) and late stationary phase (O.D. ∼1.7) cultures of MGAS2221 using rifampicin (1 mg/ml final concentration) as previously described[20]. Samples were taken before (T = 0) and after (T = 5, 10, 20, 30, 45, 60, and 90 min) rifampicin treatment. Samples were added to 2 volumes of RNA protect to prevent further RNA degradation, with GAS pelleted by centrifugation, quick frozen in liquid nitrogen, and stored at −80°C. Total RNA was isolated and subjected to Northern blot analysis.

### Construction of isogenic *pel* mutant strains

Isogenic *pel* mutants of parental strains MGAS2221, MGAS5005, MGAS5406 and MGAS9127 were constructed by replacement of *pel* with a spectinomycin resistance cassette. The strategy used to construct the mutant strains is based upon a previously described method [21]. PCR primers used in the construction of mutant strains are listed in Table S2. Confirmation of isogenic mutant strain construction was gained via PCR, sequencing, and Southern blot analyses (data not shown).

### Microarray analysis of GAS gene expression

Genome-wide analysis of GAS gene expression was achieved through use of a custom Affymetrix microarray that contained 16 antisense oligonucleotide probe pairs (PM + MM) for each gene in the MGAS2221 genome. Strains were grown in triplicate at 37°C (5% CO_2_) in THY broth. Samples were gained at mid-exponential (O.D._600_ ∼0.5) and stationary (O.D._600_ ∼1.7) phases of growth. Total RNA was isolated, converted to cDNA, labeled, and each sample hybridized to a custom array as described[19]. Gene expression estimates were calculated using GCOS software v1.4 (Affymetrix Inc.). Data were normalized across samples to minimize discrepancies that can arise due to experimental variables (e.g., probe preparation, hybridization). Genes with expression values below 100 were manually removed from the data and a two-sample *t*-test (unequal variance) applied using the statistical package Partek Pro v5.1 (Partek, Inc.). All data is MIAME compliant and the raw data has been deposited at the MIAME compliant Gene Expression Omnibus (GEO) database at National Center for Biotechnology Information (http://www.ncbi.nlm.nih.gov/geo) and are accessible through accession number GSE17790.

### Quantitative RT-PCR verification of expression microarray data

TaqMan quantitative RT-PCR was performed using an ABI 7500 Fast System (Applied Biosystems). Gene transcript levels of isogenic mutant strains were compared to parental strains using the ΔΔ*C_T_* method as described[22]. TaqMan primers and probes for the genes of interest, and the internal control gene *proS*, are listed in Table S2. Samples were ran in triplicate on three separate occasions.

### Western blot analysis of in vitro grown cultures

Supernatant proteins from overnight THY broth GAS cultures were concentrated by ethanol precipitation and resuspended in SDS-PAGE loading buffer at 1/20^th^ the original volume. HRP conjugated secondary antibodies were used to detect primary antibody binding and generate signal.

## Results

### Microarray-based identification of GAS sRNAs

A previous bioinformatic search in GAS identified 42 candidate sRNAs (Table 1, method L) [23]. As this bioinformatic approach did not identify any of the three previously described GAS sRNAs (PEL, FASX, or RIVX [13], [15], [16]) this indicates that potentially significant numbers of sRNAs remain to be identified. A powerful approach to the identification of sRNAs on a genome-wide scale has been the recent use of tiling microarrays [24], [25]. Tiling microarray approaches complement bioinformatic approaches to sRNA identification due to their ability to identify sRNAs that have a propensity to be missed by bioinformatic approaches, in particular sRNAs with limited secondary structure. Thus, the unison of both tiling microarray and bioinformatic-based investigations represents a comprehensive approach to sRNA discovery [26]–[28].

**Table 1:** Candidate small regulatory RNAs identified by bioinformatic and tiling microarray approaches.

RNA name	Left nucleotide	Right nucleotide	Size (nt)	Orientation	Information and/or adjacent genes	Method
SR79100	79100	79500	400	<	Adjacent to ribosmal protein L17P and M5005_Spy_0072	M
SR125800	125800	125900	100	<	Adjacent to a sortase and M5005_Spy_0115	M
SR146132	146132	146288	157	>	Adjacent to M5005_Spy_0135 and *purA*	L
SR188392	188392	188735	343	>	Adjacent to a tRNA-specific adenosine deaminase and M5005_Spy_0180	L
SR188971	188971	189044	73	>	Adjacent to a tRNA-specific adenosine deaminase and M5005_Spy_0180	L
SR195750	195750	195870	140	<	Adjacent to putative transcription factor and a transposase	M
SR214350	214350	214500	150	>	*fasX*	M
SR237399	237399	237709	310	>	Adjacent to ribosomal protein S7P and *fus*	L
SR254481	254481	254590	109	>	Adjacent to *sufB* and a D-alanyl-D-alanine serine-type carboxypeptidase	L
SR257300	257300	257400	100	?	Adjacent to dacA2 and oppA	M
SR263982	263982	264054	72	>	Adjacent to *oppF* and a putative transposase pseudogene	L
SR271250	271250	271350	100	>	Adjacent to *comX* and a transposase	M
SR277250	277250	277350	100	>	Adjacent to a putative methyltransferase and M5005_Spy_0267	M
SR307231	307231	307572	341	<	Adjacent to a putative transposase and inner membrane protein YIDC	L
SR331095	331095	331188	93	<	Adjacent to ferrichrome transport ATP-binding protein *fhuA* and *murE*	L
SR336250	336250	336450	200	>	Adjacent to *clpP* and M5005_Spy_0329	M
SR358650	358650	358800	150	<	Adjacent to *spyA* and M5005_Spy_0352	M
SR360800	360800	361300	500	<	Adjacent to M5005_Spy_0354 and M5005_Spy_0355	M
SR396160	396160	396487	327	>	Adjacent to *silD* and M5005_Spy_0402	L
SR418861	418861	419103	242	>	Adjacent to M5005_Spy_0426 and *thrS*	L
SR452230	452230	452500	270	>	Adjacent to M5005_Spy_0460 and M5005_Spy_0461	M
SR520921	520921	521058	138	>	Adjacent to ftsX and M5005_Spy_0533	L
SR540686	540686	540783	98	<	Adjacent to M5005_Spy_0550 and rplS	L
SR541600	541600	541800	200	>	Adjacent to a tRNA-Arg and M5005_Spy_0552	L,M
SR559590	559590	559700	110	>	*pel*	M
SR622408	622408	622534	126	>	Adjacent to *cmk* and *infC*	L
SR638450	638450	638600	150	>	Adjacent to ribosomal protein L27P and a putative transcription factor	M
SR641213	641213	641345	132	>	Adjacent to M5005_Spy_0638 and *pyrR*	L
SR678133	678133	678532	399	>	Adjacent to M5005_Spy_0674 and M5005_Spy_0676	L
SR701500	701500	702300	800	>	Adjacent to *punA* and *deoD2*	M
SR721150	721150	721350	200	?	Adjacent to tRNA-Arg and M5005_Spy_0716	M
SR758876	758876	758976	100	>	Adjacent to *acoL* and *hylA*	L
SR759205	759205	759368	163	<	Adjacent to *acoL* and *hylA*	L
SR800747	800747	800916	169	>	Adjacent to *thdF* and *rplJ*	L
SR801894	801894	802142	249	>	Adjacent to rplL and M5005_Spy_0797	L
SR843321	843321	843412	91	>	Adjacent to *pta* and M5005_Spy_0852	L
SR862600	862600	862900	300	>	Adjacent to an ABC transporter and *nox*	M
SR869300	869300	869600	300	>	Adjacent to a glyoxalase family protein and M5005_Spy_0877	M
SR914400	914400	914600	200	<	Adjacent to *rnhB* and a cardiolipin synthetase	L,M
SR933600	933600	934150	550	<	Adacent to cdd and the 16S rRNA methyltransferase	M
SR961800	961800	962000	200	<	Adjacent to M5005_Spy_0976 and *pcrA*	M
SR969000	969000	969100	100	?	Adjacent to *cfa* and a histidine-binding protein	M
SR1016300	1016300	1016500	200	>	Prophage-encoded	M
SR1018400	1018400	1018500	100	>	Prophage-encoded	M
SR1131900	1131900	1132100	200	<	Adjacent to *prfC* and *deaD*	L,M
SR1173300	1173300	1173350	50	>	Prophage-encoded	M
SR1175500	1175500	1175800	300	>	Prophage-encoded	M
SR1175900	1175900	1176100	200	>	Prophage-encoded	M
SR1201244	1201244	1201471	227	<	Adjacent to *ileS* and *divIVAS*	L
SR1207340	1207340	1207537	198	>	Adjacent to ftsA and divIB	L
SR1251900	1251900	1252100	200	<	Adjacent to M5005_Spy_1295 and a ribosomal protein	L,M
SR1291775	1291775	1291982	207	<	Adjacent to M5005_Spy_1324 and ribosome associated factor Y	L
SR1355150	1355150	1355250	100	<	Adjacent to *glpK* and M5005_Spy_1382	M
SR1358431	1358431	1358497	66	<	Adjacent to *glyS* and *glyQ*	L
SR1385110	1385110	1385204	94	<	Adjacent to M5005_Spy_1413 and bacteriophage gene M5005_Spy_1414	L
SR1532800	1532800	1532900	100	?	Adjacent to M5005_Spy_1571 and M5005_Spy_1572	M
SR1568180	1568180	1568273	93	<	Adjacent to pyrG and rpoE	L
SR1587818	1587818	1588167	349	<	Adjacent to hsdM and transcriptional regulatory protein degU	L
SR1604140	1604140	1604210	70	<	Adjacent to *lacR.2* and *dinJ*	M
SR1605828	1605828	1606280	452	<	Adjacent to an integrase pseudogene and SSU ribosomoal protein rpsI	L
SR1678800	1678800	1678950	150	<	Adjacent to *scpA* and *a transposase*	L,M
SR1678950	1678950	1679050	100	<	Adjacent to *scpA* and *a transposase*	M
SR1681917	1681917	1682067	150	>	Adjacent to virulence factors *sic* and *emm*	L
SR1698200	1698200	1698640	440	?	Adjacent to *speB* and M5005_Spy_1736	M
SR1719800	1719800	1719900	100	<	Adjacent to *pepD* and M5005_Spy_1759	M
SR1720792	1720792	1720852	60	>	Adjacent to putative transcription factor M5005_Spy_1760 and groEL	L
SR1720816	1720816	1720922	106	<	Adjacent to putative transcription factor M5005_Spy_1760 and groEL	L
SR1727893	1727893	1728188	295	<	Adjacent to a tranposase and peroxiredoxin gene ahpC	L
SR1745900	1745900	1746000	100	>	Adjacent to a putative transcriptional regulator and *rpsB*	M
SR1754950	1754950	1755050	100	<	Adjacent to *treR* and M5005_Spy_1786	L,M
SR1765900	1765900	1766000	100	?	Adjacent to *recA* and *spxA*	M
SR1789300	1789300	1789400	100	?	Adjacent to a putative transcriptional regulator and M5005_Spy_1821	L,M
SR1806601	1806601	1806858	257	<	Adjacent to *trmU* and M5005_Spy_1839	L
SR1808413	1808413	1808633	220	<	Adjacent to *trmU* and *sdhB*	L
SR1811574	1811574	1811651	77	<	Adjacent to M5005_Spy_1843 and ABC permease protein cbiQ	L

Nucleotide coordinates and gene designations are relative to the publically available MGAS5005 genome sequence [17]. Candidate sRNAs without a clearly defined orientation are highlighted with a question mark. RNAs were identified from a previous bioinformatic analysis (L) or by the microarray-based method described here (M).

**Figure 1:**
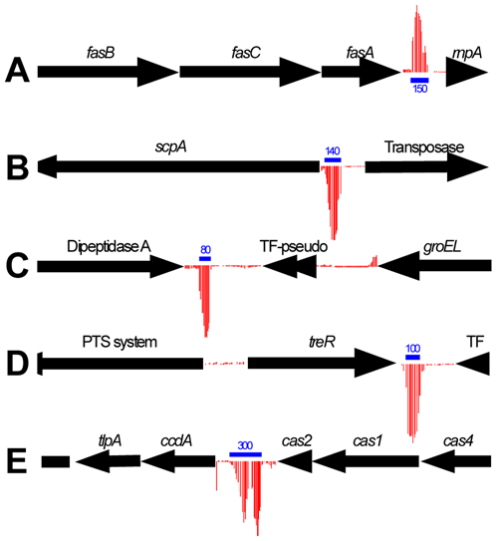
Representative candidate small RNA molecules identified by tiling microarray. Genes are represented by black arrows facing the direction of transcription. Red vertical lines represent signal intensities from probes (PM-MM) tiled within intergenic regions. Red lines extending upward indicate left to right transcription, downward extending lines indicate right to left transcription. Blue horizontal bars indicate RNA length with the size in nucleotides shown. (**A**) Validation of our custom microarray as a tool to identify GAS sRNAs. The previously described FASX sRNA is located downstream of *fasA* (M5005_spy_0206 from the published MGAS5005 genome) and can be visualized as a distinct peak of signal intensity. (**B**) A candidate sRNA located upstream, and in the same orientation as, the C5a peptidase encoding gene *scpA* (M5005_spy_1715). (**C**) A candidate sRNA located downstream, and in opposite orientation to, dipeptidase A (M5005_spy_1758). (**D**) A candidate sRNA located downstream of, and in opposite orientation to, the *treR* gene encoding a putative repressor of the trehalose operon (M5005_spy_1785). (**E**) A clustered, regularly interspaced short palindromic repeat (CRISPR) element in GAS is transcribed in the same orientation as CRISPR-associated genes (*cas1, cas2, cas4*; M5005_spy_1285-7).

To facilitate identification of candidate sRNAs transcribed by the serotype M1 GAS strain MGAS2221 we designed a custom Affymetrix microarray. The custom array consisted of overlapping 25mer oligonucleotides tiled at high density from both strands of intergenic regions within the MGAS2221 genome, with an average of 17 nucleotides of overlap between adjacent probes. Total RNA was isolated from triplicate MGAS2221 cultures during the exponential phase of growth in THY broth, converted to cDNA, labeled, and hybridized to our custom array as described in the Materials and Methods section. Candidate sRNAs were detected based upon (a) statistically significant signal intensities between PM and MM probes located within a sliding window 81 nucleotides in length (*P*<0.05, Wilcoxon signed rank test); (b) a signal intensity score >500 for at least 6 contiguous probes; and (c) visualization of signal intensities in context of genome location to eliminate signal from apparent mRNA 5′ or 3′ untranslated regions. Analysis of the resultant data indicated the presence of 40 sRNAs in the MGAS2221 genome (Figure 1 and Table 1, method M). Importantly, and in contrast to the previous bioinformatic analysis, the previously described sRNAs PEL and FASX were both identified by the tiling microarray approach (Figure 1A and data not shown), indicating that this is a powerful tool with which to identify GAS sRNAs. It should be noted that our inability to observe the sRNA RIVX in the array data was expected given the very low level of RIVX transcription by wild-type GAS strains [16]. Only 7 of the candidate sRNAs identified by microarray were also identified by the bioinformatic approach. Thus, combining bioinformatic and array data a total of 75 unique candidate sRNAs are predicted to reside within the MGAS2221 genome.

### Riboswitches and other small RNA molecules

We also identified 13 candidate small RNA molecules with proposed activities distinct from sRNAs (Table 2). Based upon sequence homology and genome location at least seven small RNAs are predicted riboswitches. Riboswitches are structures located in the 5′ region of mRNAs that can directly bind intracellular metabolites, regulating the transcription and/or translation of the downstream mRNA [29]. A microarray signal was detected from the two clustered, regularly interspaced short palindromic repeat (CRISPR) elements within the MGAS2221 genome (Figure 1E and Table 2). CRISPR elements, in association with a conserved set of genes, provide a barrier to horizontal gene transfer [30].

**Table 2:** Candidate riboswitches and other small RNAs identified by bioinformatic and tiling microarray approaches.

RNA name	Left nucleotide	Right nucleotide	Size (nt)	Orientation	Information and/or adjacent genes
RNA190950	190950	191010	60	**>**	Putative SRP RNA
RNA319780	319780	319900	120	**>**	Putative riboswitch
RNA484784	484784	484936	152	**<**	Putative vitamin B1 riboswitch
RNA501950	501950	502050	100	**>**	Putative *pacL* riboswitch
RNA772970	772970	773110	140	**<**	CRISPR - 1
RNA849201	849201	849250	49	**>**	Putative purine riboswitch
RNA964800	964800	964850	50	**>**	Putative glycine riboswitch
RNA983400	983400	983800	400	**>**	Putative tmRNA
RNA1083000	1083000	1083250	250	**<**	Putative *metK2* riboswitch
RNA1239700	1239700	1240020	320	**<**	CRISPR - 2
RNA1320100	1320100	1320500	400	**<**	Putative RNase P
RNA1439100	1439100	1439200	100	**>**	Putative *serS* riboswitch
RNA1660600	1660600	1660800	200	**>**	Putative 6S RNA

Nucleotide coordinates and gene designations are relative to the publically available MGAS5005 genome sequence [17].

### Northern blot verification of sRNA transcription

**Figure 2:**
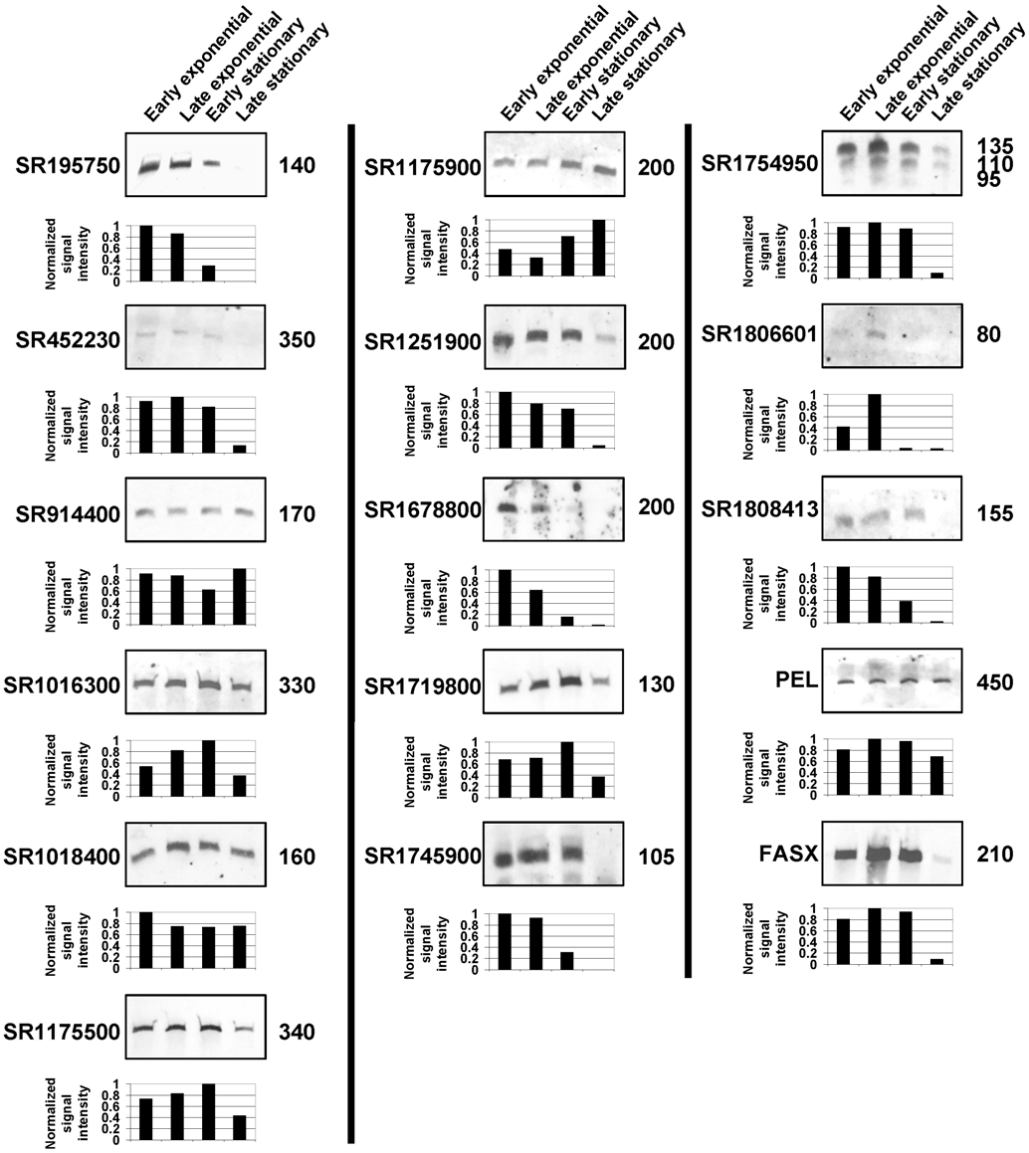
Northern blot verification of candidate sRNAs. Northern blots were performed using RNA isolated from strain MGAS2221 at 4 growth phases and probed for the presence of candidate sRNAs. The name or genome location (in nucleotides, relative to the published MGAS5005 genome) of candidate RNAs is displayed to the left of each blot. The approximate size in nucleotides of detected transcript/s is displayed to the right of each blot. Below each blot is a graph representing the normalized signal intensity of each hybridizing band. Signal intensities were generated using the Quantity One software package version 4.6.1., and normalized to signal detected for the housekeeping RNA 5S RNA (a representative 5S RNA blot is shown in figure 3). Normalized signal intensities are plotted relative to the most highly expressed time-point.

To verify that sRNAs are transcribed at the locations indicated by bioinformatic and microarray analyses we performed Northern blot analysis. A total of 32 candidate sRNAs were tested by Northern analysis, and were selected primarily from those candidates identified by the microarray approach (see Table S1). We observed a transcript for 16 out of the 32 candidate sRNAs tested (Figure 2). Several of the candidate sRNAs showed variation in transcript concentration during growth, with transcripts decreasing in abundance during stationary phase in most cases (Figure 2). While we are unable to state that these sRNAs are transcribed in a growth-phase dependent manner due to the potential degradation of sRNAs by ribonucleases at specific growth phases, we can state that they show growth-phase dependent regulation of RNA abundance, a function of both RNA synthesis and decay [20].

**Figure 3:**
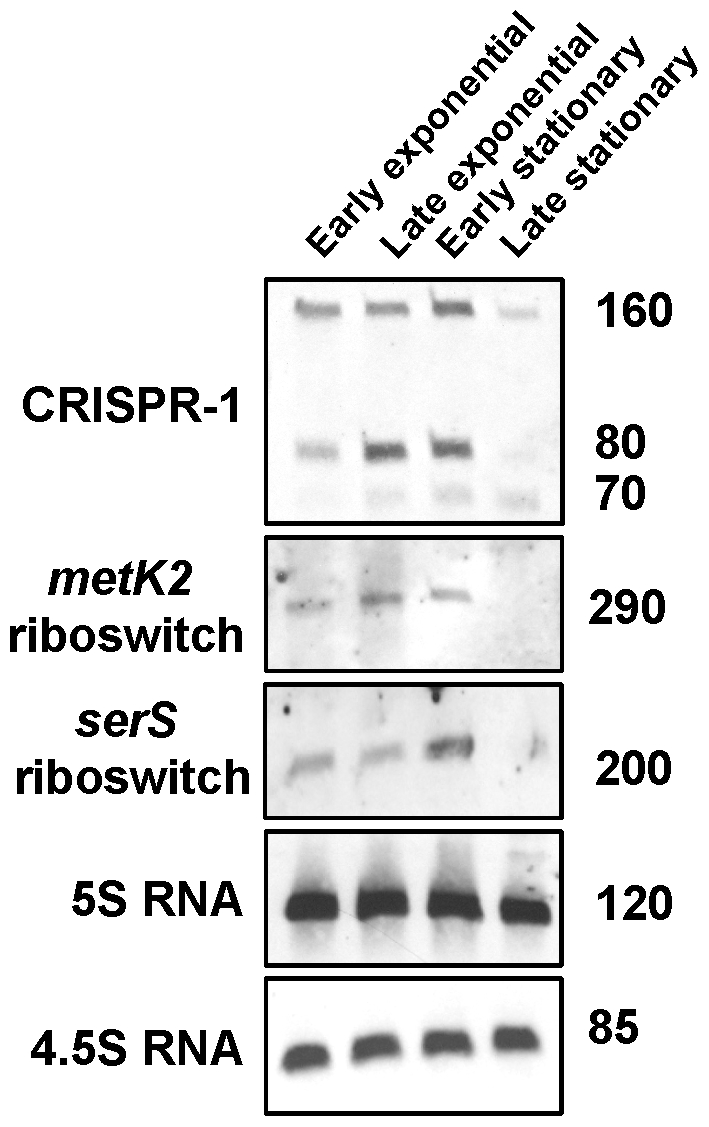
Northern blot verification of riboswitches and other small RNAs. Northern blots were performed using RNA isolated from strain MGAS2221 at 4 growth phases. The name of the candidate RNA molecules are shown to the left of each Northern. To the right of each Northern is the approximate size in nucleotides of the transcript/s. The 5S RNA served as a loading control.

Small RNA molecules corresponding to the 4.5S RNA, *metK2* riboswitch, *serS* riboswitch and CRISPR-1 element were also probed by Northern blot (Figure 3). The 4.5S RNA represents the RNA component of the signal recognition particle (SRP) that facilitates protein secretion via the co-translational pathway [31]. Given the important function of the SRP pathway it is not surprising that the 4.5S RNA is transcribed throughout growth [32]. The *metK2* and *serS* riboswitches, based upon analogies to the function of these riboswitches in other organisms, should decrease transcription of their corresponding genes in the presence of SAM and charged seryl-tRNAs, and increase transcription of these genes in the absence of SAM and charged seryl-tRNAs, respectively. The small RNAs identified by Northern for the two riboswitches presumably represent transcription termination products, with termination occurring during exponential phase where SAM and charged seryl-tRNAs are not limiting (Figure 3). CRISPR elements are transcribed as single transcripts and subsequently processed into smaller RNA molecules [30], a fact that is consistent with our observation of a multiple banding pattern for GAS CRISPR-1 transcripts (Figure 3).

### sRNA gene and promoter analysis

**Figure 4:**
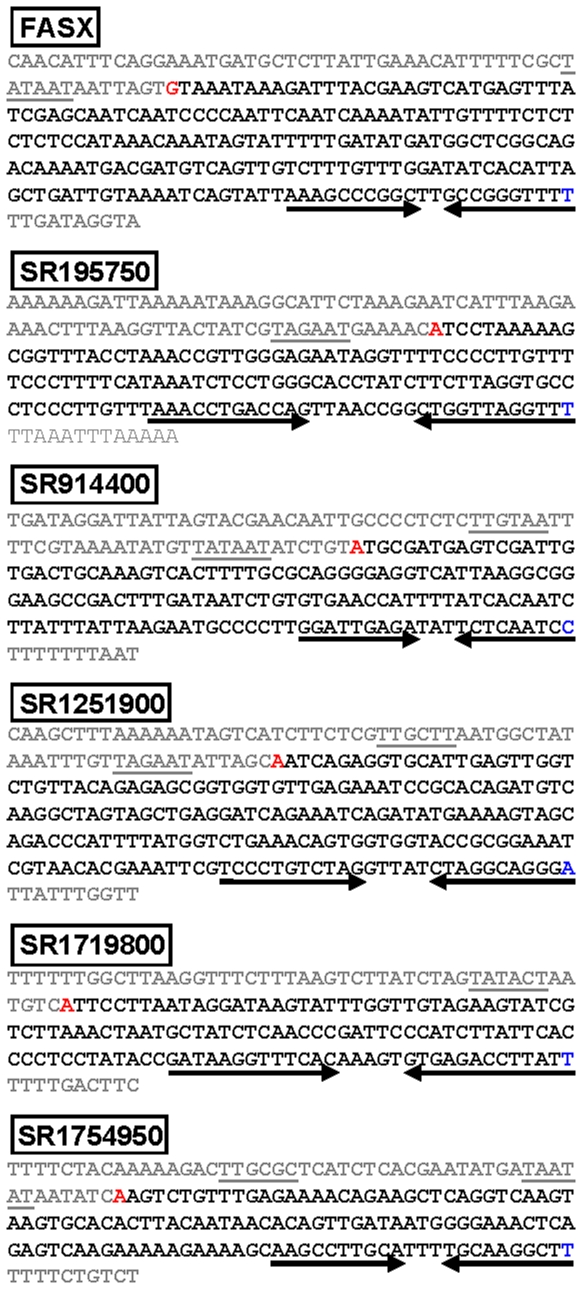
Analysis of candidate sRNA transcriptional start sites, terminators, and promoter regions. The transcriptional start sites of candidate sRNAs FASX, SR195750, SR914400, SR1251900, SR1719800, and SR1754950 were determined by 5′ RACE. The identified transcriptional start site is colored red, the deduced sRNA sequences are colored black, and the final base of the terminator hairpin is colored blue. The putative −10 and/or −35 promoter sequences are underlined and putative rho-independent (intrinsic) terminators are highlighted by inverted arrows.

We selected six candidate sRNAs and determined their sequence by measuring the approximate length of the transcripts via Northern blot analysis (Figure 2), identifying the sRNA transcriptional start sites via 5′ rapid amplification of cDNA ends (5′ RACE) [33], and using the transcriptional start site and transcript length data to identify putative transcriptional terminators (Figure 4). As most sRNAs function through a process involving complementary base-pairing with target mRNA molecules, the deduced sequence of these sRNAs may facilitate the identification of putative mRNA targets, for example by using the sRNA sequence data in a bioinformatic program such as TargetRNA [34]. Analysis of the promoter regions of the six sRNAs identified no shared sequence motifs.

### Analysis of sRNA stability

**Figure 5:**
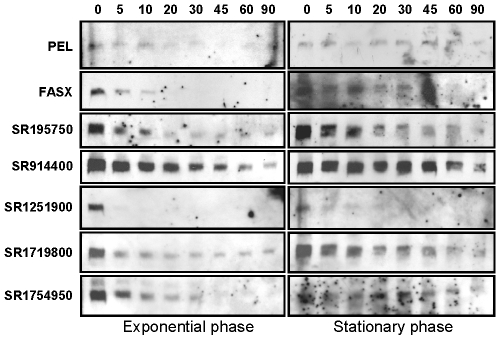
Northern blot analysis of sRNA stability. Aliquots of mid-exponential or late stationary phase cultures of strain MGAS2221 were harvested prior to (T = 0) and following (T = 5, 10, 20, 30, 45, 60, 90 min) rifampicin treatment to inhibit new RNA synthesis. 8 µg of extracted RNA from each time-point was subjected to Northern blot analysis, probing for PEL, FASX, SR195750, SR914400, SR1251900, SR1719800, and SR1754950 transcripts. Note that as the exposure time of each Northern blot varied no comparison of band intensities between blots should be made.

The abundance of any sRNA at a given time point is a reflection of the rate of its synthesis and decay. To measure the stability of candidate sRNAs we performed Northern blot analysis using RNA isolated from GAS cultures that were treated with rifampicin to inhibit RNA synthesis. All seven of the sRNAs tested were more stable during stationary phase than exponential phase (Figure 5), in keeping with data from a previous study that measured mRNA stability [20]. Given that the sRNAs tested were generally more abundant during exponential phase than stationary phase (Figure 2), the apparent reduced rate of sRNA transcription in stationary phase more than offsets any influence on sRNA abundance caused by increased stability. The stability of individual sRNAs varied widely from highly stable (SR914400) to highly unstable (SR1251900), similar to that observed for sRNAs in other bacteria [35], [36].

### Analysis of strain and/or serotype-specific variation in sRNA transcription

**Figure 6:**
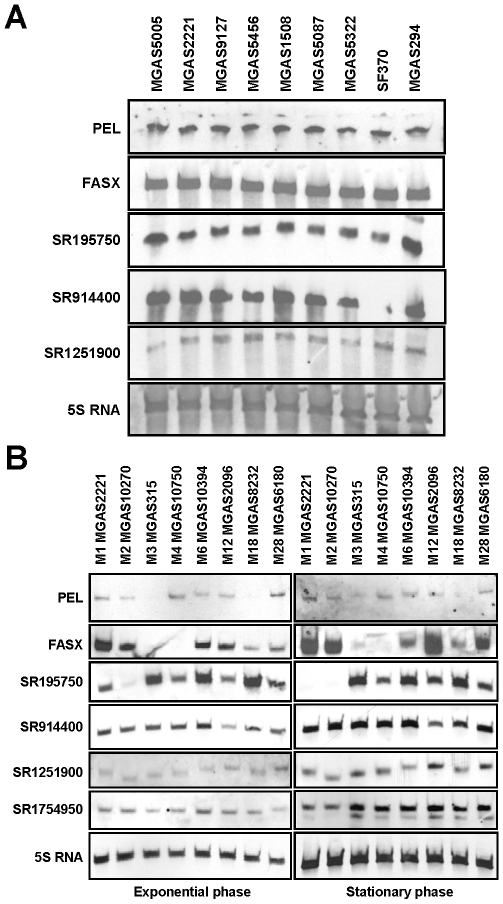
Northern blot analysis of intra- and/or inter-serotype variation in sRNA transcription. (**A**) Intra-serotype variation. Transcript abundance of sRNAs PEL, FASX, SR195750, SR914400, and SR1251900 were assayed in 9 different serotype M1 GAS strains. The M1 GAS strains were isolated from several different countries over a greater than 10 year period (Table S5). Northern blots were made using RNA isolated from exponential phase cultures. Note that an air bubble, and not a lack of transcript, was responsible for the apparent lack of signal for SR914400 in the SF370 sample. The housekeeping 5S RNA was used as a loading control. (**B**) Inter-serotype variation. Transcript abundance of sRNAs PEL, FASX, SR195750, SR914400, SR1251900, and SR1754950 were assayed in strains representing 8 GAS serotypes. Northern blots were made using RNA isolated from both exponential and early stationary phase cultures of the serotype M1 strain MGAS2221, the serotype M2 strain MGAS10270, the serotype M3 strain MGAS315, the serotype M4 strain MGAS10750, the serotype M6 strain MGAS10394, the serotype M12 strain MGAS2096, the serotype M18 strain MGAS8232, and the serotype M28 strain MGAS6180. The housekeeping 5S RNA was used as a loading control.

The transcript levels of several *S. aureus* sRNAs fluctuate between clinical isolates, potentially resulting in derivatives with distinct virulence characteristics [37], [38]. We set out to assay whether sRNA transcript abundance varied within and/or between different GAS serotypes. Northern blot analysis using RNA isolated from nine serotype M1 strains identified that, with the possible exception of increased SR195750 expression in strains MGAS5005 and MGAS294, no variation in transcript abundance was observed for the five candidate sRNAs tested (Figure 6A). In contrast, comparing sRNA transcript abundance in GAS strains representing eight different serotypes we identified an apparent serotype-specific abundance for sRNAs PEL, FASX, and SR195750 (Figure 6B). RNA from the serotype M3 and M4 strains showed little to no hybridization with the FASX probe, an interesting observation given its role in virulence factor regulation [15]. Likewise, hybridization to the SR195750 probe was not observed for the M1 and M2 strains during the stationary phase of growth, while all other strains, and in particular the M3, M6, and M18 strains, exhibited abundant SR195750 transcript levels. While there was little variation in SR1251900 transcript abundance among the eight difference serotypes we did observe variation in transcript size (Figure 6B).

### Analysis of the PEL regulon in M1T1 GAS

**Figure 7:**
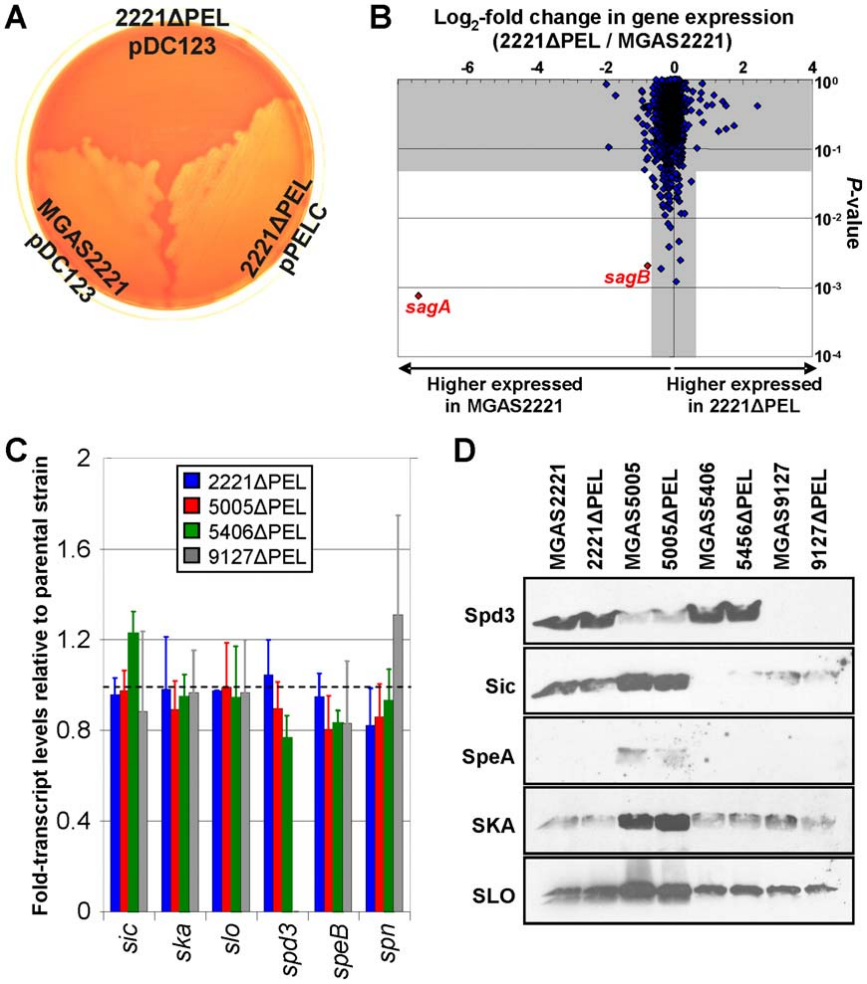
PEL has no apparent regulatory function in four M1T1 clinical GAS isolates. (**A**) Plate assay showing that the hemolytic negative phenotype of mutant strain 2221ΔPEL is complemented by addition of plasmid pPELC. Plasmid pPELC is a derivative of vector pDC123 that contains wild-type PEL. (**B**) Fold change (log_2_) in gene expression between isogenic mutant strain 2221ΔPEL and parental strain MGAS2221 during the exponential phase of growth in THY broth. Corresponding *P*-values (T-test) are graphed on the *y*-axes. The two white background areas of the graph signify those genes which are differentially expressed ≥1.5-fold with *p*≤0.05. Data points corresponding to genes of interest are colored red and labeled. (**C**) Taqman quantitative RT-PCR analyses comparing the transcript levels of select genes between parental strains MGAS2221, MGAS5005, MGAS5406, MGAS9127, and their isogenic *pel* mutant derivatives. Note that the *spd3* gene is absent in strain MGAS9127. Experiment was performed in triplicate with mean fold-transcript levels relative to the appropriate parental strain (dashed line) shown. Error bars represent ± standard deviation. (**D**) Western blot analyses showing a lack of regulation by PEL in the four M1T1 GAS isolates studied. Western blots were created using protein isolated from the supernatants of exponential phase THY cultures of each GAS strain.

The role of PEL in regulating GAS virulence gene expression has mainly been investigated by Northern blot analyses of select genes [13], [14]. To investigate PEL-mediated gene regulation on a genome-wide scale we performed expression microarray analysis. To facilitate analysis of the genes regulated by PEL in strain MGAS2221 we constructed the isogenic PEL mutant strain 2221ΔPEL. 2221ΔPEL was created using a well-described PCR-based procedure that replaced PEL with a spectinomycin resistance cassette [39]. PEL is an atypical sRNA in that it also functions as an mRNA, encoding the hemolysin streptolysin S from the *sagA* gene [13], [40]. We were able to exploit this function to confirm loss of PEL/*sagA* in strain 2221ΔPEL using a hemolysis plate assay (Figure 7A). Parental strain MGAS2221 containing vector pDC123 gave a typical β-hemolytic morphology when streaked onto agar plates containing 5% sheep blood. In contrast, isogenic mutant 2221ΔPEL containing vector pDC123 failed to show hemolytic activity (Figure 7A). Hemolytic activity was restored to 2221ΔPEL by introduction of plasmid pPELC, a pDC123 derivative containing wild-type PEL.

Expression microarray comparisons of strains MGAS2221 and 2221ΔPEL were performed using RNA isolated from triplicate cultures of each strain grown in THY broth at both the exponential and stationary phases of growth. Somewhat surprisingly, only 2 genes met our criteria of being differentially expressed (fold-change ≥1.5-fold, *P*-value≤0.05) between MGAS2221 and isogenic mutant 2221ΔPEL at either time-point (Figure 7B and data not shown). These differentially regulated genes were *sagA* encoding streptolysin S (169 and 734-fold decreased expression in 2221ΔPEL during exponential and stationary phases, respectively), and the downstream gene *sagB* encoding a protein involved in the processing and transport of streptolysin S (2 and 3-fold decreased expression in 2221ΔPEL during exponential and stationary phases, respectively) [40]. The significant down-regulation of *sagA* is due to this gene being encoded within the PEL RNA molecule [13], and hence is deleted in strain 2221ΔPEL. As some PEL/*sagA* transcripts also read-through into the downstream *sagB* gene, the deletion of PEL/*sagA* also provides an explanation for the reduction in the level of *sagB* transcripts [40].

To address whether the lack of PEL regulatory function was a common occurrence in M1T1 GAS we created three additional *pel* isogenic mutants in the M1T1 background and subjected them to quantitative RT-PCR and Western blot analyses. The three additional parental M1T1 strains differed in their year and country of isolation, and their disease characteristics (Table S5). The genes and proteins investigated by quantitative RT-PCR and Western blot were previously described as being PEL-regulated [13], [14]. Similar to the expression microarray data, we essentially observed no difference between parental and isogenic mutant strains (Figures 7C and 7D). Our data are consistent with PEL having no regulatory function in M1T1 GAS.

## Discussion

Regulating gene expression to microenvironment-specific conditions is key to the ability of bacterial pathogens to infect and cause disease. Here, we show that sRNAs are abundantly transcribed throughout the GAS genome, with 75 unique candidate sRNAs identified via our microarray-based approach and a previous bioinformatic approach [23]. As this number approximates the number of GAS transcription factors this raises the possibility that sRNA-mediated regulation represents a major mechanism of regulation in this pathogen. Indeed, as only exponential phase GAS was analyzed by tiling microarray it is possible that additional sRNAs would be discovered in GAS grown to other growth phases. While regulatory functions for the newly discovered sRNAs have yet to be shown, the observation that many show growth phase-dependent regulation of transcript abundance is consistent with these sRNAs potentially regulating expression in a growth phase-dependent manner. Our dataset should promote investigation of sRNA-mediated regulation in this important Gram-positive pathogen.

Of the 75 candidate sRNAs cumulatively identified only 7 were identified by both microarray and bioinformatic methods. As the microarray method can only detect transcribed sRNAs, and some sRNAs may only be transcribed in response to specific growth phase or *in vivo* signals, it is possible that several sRNAs currently identified only via bioinformatics will also be identified by microarray once planned *in vitro* and *ex vivo* experiments are performed. We cannot discount the possibility that some sRNAs may have been missed in our study due to mischaracterization of microarray probe signal as belonging to mRNA 5′ or 3′ untranslated regions rather than to sRNAs. The potential to mischaracterize signal intensity increases for poorly transcribed sRNAs that are located adjacent to highly transcribed mRNAs, especially if the genes are in close proximity to one-another. The bioinformatic approach, while not identifying any of the three previously described GAS sRNAs (PEL, FASX, and RIVX), did identify unique sRNAs (Table 1). Thus, while the software requires optimizing for GAS sRNA prediction, it never-the-less has been a useful tool in GAS sRNA discovery [23]. The minimal level of overlap between the microarray and bioinformatic sRNA identification methods is consistent with that observed in other studies [27], [28], and underpins the importance of a multifaceted approach to sRNA identification

Transcription of 32 of the 75 identified candidate sRNAs were tested by Northern blot analysis, of which 16 gave a hybridizing signal (Figure 2 and Table S1). The absence of a Northern hybridizing signal does not necessarily imply that a candidate sRNA is a false-positive. For example, the sRNA transcript level could be below the limit of detection of our Northern protocol, or there could be an absence of inducing signal for sRNA transcription prior to RNA isolation.

The 75 candidate GAS sRNAs show variable presence and conservation in the dozen publically available GAS genome sequences (Table S4). While 62 candidate sRNAs were present in all of the sequenced genomes tested, 13 were absent from at least one genome. Of the variably present sRNAs five were bacteriophage-encoded, with acquisition or loss of prophage being the most common mechanism explaining the variable presence of these sRNAs. Given that phage-encoded sRNAs have the potential to regulate host chromosomal genes [37], and that GAS are commonly lysogenized by multiple prophage [41], phage-encoded sRNAs may play important roles in modulating GAS gene expression.

Only minor intra-serotype variability in sRNA transcript abundance was observed in the nine serotype M1 strains analyzed by Northern blot (Figure 6A), namely a 2–3 fold higher level of SR195750 transcripts in strains MGAS5005 and MGAS294. Interestingly, MGAS5005 and MGAS294 contain natural mutations within the gene encoding the sensor kinase CovS, a protein that in conjunction with its cognate response regulator CovR, negatively regulates ∼15% of the genes in the GAS genome including many virulence factors [11], [42]–[45]. The CovR/S-mediated repression of SR195750 transcription would be consistent with the known ability of this system to repress the downstream transcription factor-encoding gene *rivR* [46].

In contrast to intra-serotype variation in sRNA transcript abundance inter-serotype variation was more pronounced (Figure 6B). The significant variation in FASX and SR195750 transcript levels among serotypes was not due to differences in sequence identity, and hence probe hybridization kinetics, as there was no correlation between percent sequence identity and Northern hybridization intensity (Table S3). Given that FASX enhances expression of the secreted virulence factors streptokinase (Ska) and streptolysin S (SLS), and reduces expression of several extracellular matrix binding proteins, the variation in FASX transcript levels among clinical isolates may impact their virulence potential [15].

Published data both supports [13], [14] and contradicts [47], [48] a role for PEL in regulating GAS virulence gene expression. While serotype-specific phenotypes have been described in GAS this cannot be the case for PEL due to the common use of serotype M1 GAS strains in these previous studies. We identified no differentially expressed genes between strains MGAS2221 and 2221ΔPEL during exponential and stationary growth other than the PEL-encoded gene *sagA* and the downstream gene *sagB* (Figure 7B). As transcripts previously described as being PEL-regulated were unchanged following PEL mutation in three additional M1T1 GAS isolates (Figure 7C), our data is consistent with PEL having no regulatory activity in isolates of the globally disseminated M1T1 clone [17], [18], at least not under the conditions tested. Our data however must be reconciled with that from Li and colleagues who found a regulatory phenotype in an M1T1 strain transduced with a PEL transposon mutation [14]. As the transposon was transduced into the M1T1 strain from an M49 strain it is possible that sequences adjacent to the transposon were also transduced, and that these sequences are responsible for the observed phenotype. Possible support for this hypothesis is that the passage of a *pel* transposon mutant through mice resulted in restoration of *pel* transcription even though the transposon remained inserted upstream of *pel* [49]. If PEL-mediated regulation does occur in M1T1 GAS in a strain-specific manner then only one or a small number of genetic changes must account for whether PEL has regulatory activity as M1T1 GAS strains have highly similar genomes (e.g. M1T1 strains MGAS5005 and MGAS2221 have only 20 genetic differences [mostly single nucleotide polymorphisms] between them despite being isolated on different continents eight years apart [11]).

The ability of GAS to cause a wide variety of diseases is in part due to the coordinate expression of specific subsets of virulence factors in response to microenvironment-dependent stimuli. While not yet proven, the discovery of sRNA transcripts transcribed throughout the genome raises the possibility that sRNA-mediated regulation has a greater role in controlling GAS gene expression than previously recognized. Based upon the estimated number of sRNAs within bacterial genomes a total of 75 candidate sRNAs places GAS in the middle of those bacteria analyzed, with approximately an order of magnitude less sRNAs than *E. coli* and an order of magnitude more than *Borrelia burgdorferi* [27], [28], [50]. The data presented in this manuscript provides a significant resource for future investigations of sRNAs and their role in regulating the virulence of GAS and related pathogens.

## Supporting Information

Table S1 Distribution across discovery method for candidate sRNAs selected for Northern analysis. Thirty two candidate sRNAs were selected for Northern analysis. Selected sRNAs were originally identified by our tiling microarray approach (M) and/or a previous bioinformatic approach (L) [22].
(0.06 MB DOC)

Table S2 Primers and probes used in this study.
(0.14 MB DOC)

Table S3 Percent identity between strains of probes used in the serotype Northern blots.
(0.07 MB DOC)

Table S4 Percent conservation of candidate sRNAs across the 12 sequenced GAS strains. We report percent conservation as a measure of percent identity multiplied by the percent coverage.
(0.20 MB DOC)

Table S5 Serotype M1 GAS strains studied.
(0.07 MB DOC)
