# Supplementary material for: A Genome-Wide Analysis of Small Regulatory RNAs in the Human Pathogen Group A Streptococcus
Source: PLoS One. 2009 Nov 2;4(11):e7668. doi: 10.1371/journal.pone.0007668 (PMC2765633; doi:10.1371/journal.pone.0007668)
Supplement: Table S1 — Distribution across discovery method for candidate sRNAs selected for Northern analysis. Thirty two candidate sRNAs were selected for Northern analysis. Selected sRNAs were originally identified by our tiling microarray approach (M) and/or a previous bioinformatic approach (L) [22]. (0.06 MB DOC) [file pone.0007668.s001.doc]

**Table S1**

|  | M+L | M | L |
| --- | --- | --- | --- |
| Number of sRNA candidates tested | 6 | 23 | 3 |
| Number that gave signal on Northern | 4 | 10 | 2 |
| Number that gave no signal on Northern | 2 | 13 | 1 |
