# Supplementary material for: A Genome-Wide Analysis of Small Regulatory RNAs in the Human Pathogen Group A Streptococcus
Source: PLoS One. 2009 Nov 2;4(11):e7668. doi: 10.1371/journal.pone.0007668 (PMC2765633; doi:10.1371/journal.pone.0007668)
Supplement: Table S2 — Primers and probes used in this study. (0.14 MB DOC) [file pone.0007668.s002.doc]

**Table S2**

| **Name** | **Sequence (5' - 3')** | **Information** |
| --- | --- | --- |
| PELA | GATAAAGGTATTACTGCTATCC | Primer to KO PEL |
| PELB | GTTATAGTTATTATAACATGTATTGTAAAAAATGATTAATATGTAAACCCTTTC | Primer to KO PEL |
| PELC | CTATTTAAATAACAGATTAAAAAAATTATAAGGTGTTAGAAAACATGAGACAAAAGTAATC | Primer to KO PEL |
| PELD | GTGTATTTTTCATGTAATGGTAG | Primer to KO PEL |
| PELSF | GAAAGGGTTTACATATTAATCATTTTTTACAATACATGTTATAATAACTATAAC | Primer to KO PEL |
| PELSR | GATTACTTTTGTCTCATGTTTTCTAACACCTTATAATTTTTTTAATCTGTTATTTAAATAG | Primer to KO PEL |
| PROSTMF | GCTGACCGCAAAGTGCAAA | Taqman primer |
| PROSTMR | TGACTCCTGTCATATGGAAACCA | Taqman primer |
| PROSTMP | TACCAATGCCGTTGCAGGAGCTAACAA | Taqman probe |
| SICTMF | CTGAGGACACCCCTCGTTTC | Taqman primer |
| SICTMR | TCTTGTGGATTTTTTTGAGGAGTATG | Taqman primer |
| SICTMP | TGAACCTCGTGTGACAGAAAAACCGCA | Taqman probe |
| SKATMF | CGGCTACTTTGAGGTCATTGATT | Taqman primer |
| SKATMR | CCGAACCATCTTTGTCAGCAA | Taqman primer |
| SKATMP | CAAGCGATGCAACCATTACTGATCGAAAC | Taqman probe |
| SLOTMF | GACCTTTAAAGAGTTGCAACGAAAA | Taqman primer |
| SLOTMR | GACCATAAGCTACGTTACTCACAAAGA | Taqman primer |
| SLOTMP | TGTCAGCAATGAAGCCCCGCC | Taqman probe |
| SPD3TMF | GGCGATCTTGACAATCTGCAA | Taqman primer |
| SPD3TMR | CAGCCAGGAGGATTGAATTTAAGT | Taqman primer |
| SPD3TMP | CCAACCTTCGCACACATCCAGCTAAAAG | Taqman probe |
| SPEBTMF | TGCAGGTAGCTCTCGTGTTCA | Taqman primer |
| SPEBTMR | TGCTTCCCAATCTTGTTTGCT | Taqman primer |
| SPEBTMP | ATCTGTTCACCAAATCAACCGTGGCG | Taqman probe |
| SPNTMF | AACGCAACTTCCTCTAAAGAAGACA | Taqman primer |
| SPNTMR | TGAATTTTCCTCCCAGACAGTACTC | Taqman primer |
| SPNTMP | TCATGTCATGCACACATTAGACGGCTCA | Taqman probe |
| SR195750F | CTTAATACGACTCACTATAGGGAGACCTAACCAGCCGGTTAAC | Northern probe primer |
| SR195750R | CATCCTAAAAAGCGGTTTACC | Northern probe primer |
| SR452230F | GTTATATTGGGAGTTGAGATG | Northern probe primer |
| SR452230R | CTTAATACGACTCACTATAGGGAGTTATCGCTCGTTCTTCAAGC | Northern probe primer |
| SR914400F | CTTAATACGACTCACTATAGGGAGGGGCATTCTTAATAAATAAG | Northern probe primer |
| SR914400R | TATCTGTATGCGATGAGTCG | Northern probe primer |
| SR1016300F | AGCTATCATTACGAAAGGAG | Northern probe primer |
| SR1016300R | CTTAATACGACTCACTATAGGGAGACGTCAACGGTTTCGTTGTG | Northern probe primer |
| SR1018400F | AGGGGGAGAGGACTTCTTAG | Northern probe primer |
| SR1018400R | CTTAATACGACTCACTATAGGGAGTAAACGCAAAGAACTAGAAGTCG | Northern probe primer |
| SR1175500F | GCTGATGTTATTGTTAGCAG | Northern probe primer |
| SR1175500R | CTTAATACGACTCACTATAGGGAGTGATTAGCTCTCTGCCCATC | Northern probe primer |
| SR1175900F | CAAAAAAGTACGCTATCCAATC | Northern probe primer |
| SR1175900R | CTTAATACGACTCACTATAGGGAGCACTAATCAGCACTGTCATC | Northern probe primer |
| SR1251900F | CTTAATACGACTCACTATAGGGAGAATAAGCCATCATCTTCTTTC | Northern probe primer |
| SR1251900R | GCATTGAGTTGGTCTGTTAC | Northern probe primer |
| SR1678800F | CGATTCTCTCCTAGCTGTCC | Northern probe primer |
| SR1678800R | GCTGACCTTTACTAATAATC | Northern probe primer |
| SR1719800F | CTCACACTTTGTGAAACCTTATC | Northern probe primer |
| SR1719800R | CTTATCTAGTATACTAATGTCATTCC | Northern probe primer |
| SR1745900F | TAAACAAAGAAGCTAGTCATCTC | Northern probe primer |
| SR1745900R | CTTAATACGACTCACTATAGGGAGTCTTCCAGACTTCGACTTGC | Northern probe primer |
| SR1754950F | CTTGCTTTTCTTTTTCTTGACTC | Northern probe primer |
| SR1754950R | AAGACTTGCGCTCATCTCAC | Northern probe primer |
| SR1806601F | CTTAATACGACTCACTATAGGGAGCTCATTTCTAGAGCTAAACC | Northern probe primer |
| SR1806601R | TCGCCTCGCTAGATTAGAC | Northern probe primer |
| SR1808413F | CTTAATACGACTCACTATAGGGAGATAGAAAAAGATAATCATC | Northern probe primer |
| SR1808413R | AACAAAAAAATATCAAAAAACAGC | Northern probe primer |
| PELF | GATAGTTGTTGTGTTACAACAG | Northern probe primer |
| PELR | CTTAATACGACTCACTATAGGGAGTAACTGATAAGAACGCGAG | Northern probe primer |
| FASXF | GAAGTCATGAGTTTATCGAG | Northern probe primer |
| FASXR | CAAACAAAGACAACTGACATCG | Northern probe primer |
| CRISPR1F | CCACTTTTTCAAGTTGATAAC | Northern probe primer |
| CRISP1R | CTGATAAATTTCTTTGAATTTCTCC | Northern probe primer |
| METK2F | CTTAATACGACTCACTATAGGGAGCCAAAACCAACTTAGCTAATC | Northern probe primer |
| METK2R | ATAGATTGTTATTGAGTCCC | Northern probe primer |
| SERSF | GTTATAATAGTCCTAACGGATAAG | Northern probe primer |
| SERSR | CTTATTACGACTCACTATAGGGAGCTTATGAAGATGCTGGAGCG | Northern probe primer |
| 5SF | GTTAAGTGACGATAGCCTAG | Northern probe primer |
| 5SR | CTTAATACGACTCACTATAGGGAGCTAAGCGACTACCTTATCTC | Northern probe primer |
| 4.5SF | CTTGCAAACTGTCCGTAATATG | Northern probe primer |
| 4.5SR | CTTAATACGACTCACTATAGGGAGGAAATTACTTTAGTAAAG | Northern probe primer |
| RIVRF | GAACATCTCAAATCCTTGCC | Northern probe primer |
| RIVRR | CTTAATACGACTCACTATAGGGAGCAGAACTAATACTGCCTTGAC | Northern probe primer |
| FASXGSP1 | TTACAATCAGCTAATGTG | 5' RACE primer |
| FASXGSP2 | CAAACAAAGACAACTGACATC | 5' RACE primer |
| 195750GSP1 | AACTGGTCAGGTTTAAAC | 5' RACE primer |
| 195750GSP2 | GGCACCTAAGAAGATAGGTG | 5' RACE primer |
| 914400GSP1 | AATAAATAAGATTGTG | 5' RACE primer |
| 914400GSP2 | AAATGGTTCACACAGATTATC | 5' RACE primer |
| 1251900GSP1 | CCCTGCCTAGATAACC | 5' RACE primer |
| 1251900GSP2 | TAGACAGGGACGAATTTCGTG | 5' RACE primer |
| 1719800GSP1 | ATAGGAGGGGTGAATAAG | 5' RACE primer |
| 1719800GSP2 | TGGGAATCGGGTTGAGATAG | 5' RACE primer |
| 1754950GSP1 | GCTTTTCTTTTTCTTGAC | 5' RACE primer |
| 1754950GSP2 | CTGAGTTTCCCCATTATCAAC | 5' RACE primer |
