# Supplementary material for: A Genome-Wide Analysis of Small Regulatory RNAs in the Human Pathogen Group A Streptococcus
Source: PLoS One. 2009 Nov 2;4(11):e7668. doi: 10.1371/journal.pone.0007668 (PMC2765633; doi:10.1371/journal.pone.0007668)
Supplement: Table S3 — Percent identity between strains of probes used in the serotype Northern blots. (0.07 MB DOC) [file pone.0007668.s003.doc]

Table S3

| sRNA | MGAS5005 | MGAS10270 | MGAS315 | MGAS10750 | MGAS10394 | MGAS2096 | MGAS8232 | MGAS6180 | Probe size | Comments |
| --- | --- | --- | --- | --- | --- | --- | --- | --- | --- | --- |
|  | M1 | M2 | M3 | M4 | M6 | M12 | M18 | M28 |  |  |
| SR195750 | 100 | 100 | 98 | 100 | 100 | 100 | 100 | 98 | 139 |  |
| SR914400 | 100 | 100 | 100 | 100 | 100 | 100 | 99 | 100 | 129 |  |
| SR1251900 | 100 | 99 | 95 | 95 | 95 | 99 | 95 | 95 | 222 | SNPs concentrated in terminator |
| SR1754950 | 100 | 100 | 100 | 100 | 100 | 100 | 99 | 99 | 141 |  |
| FasX | 100 | 100 | 100 | 100 | 100 | 99 | 100 | 100 | 132 |  |
| PEL | 100 | 99 | 99 | 100 | 99 | 100 | 97 | 100 | 108 |  |
| 5S RNA | 100 | 100 | 100 | 100 | 100 | 100 | 100 | 100 | 116 |  |
