# Supplementary material for: A Genome-Wide Analysis of Small Regulatory RNAs in the Human Pathogen Group A Streptococcus
Source: PLoS One. 2009 Nov 2;4(11):e7668. doi: 10.1371/journal.pone.0007668 (PMC2765633; doi:10.1371/journal.pone.0007668)
Supplement: Table S4 — Percent conservation of candidate sRNAs across the 12 sequenced GAS strains. We report percent conservation as a measure of percent identity multiplied by the percent coverage. (0.20 MB DOC) [file pone.0007668.s004.doc]

**Table S4**

|  | **MGAS5005** | **SF370** | **MGAS10270** | **MGAS315** | **SSI-1** | **MGAS10750** | **Manfredo** | **MGAS10394** | **MGAS2096** | **MGAS9429** | **MGAS8232** | **MGAS6180** |
| --- | --- | --- | --- | --- | --- | --- | --- | --- | --- | --- | --- | --- |
|  | **M1** | **M1** | **M2** | **M3** | **M3** | **M4** | **M5** | **M6** | **M12** | **M12** | **M18** | **M28** |
| SR79100 | 100 | 100 | 99 | 97 | 97 | 98 | 98 | 98 | 98 | 98 | 99 | 98 |
| SR125800 | 100 | 100 |  |  |  |  |  | 100 |  |  | 0 | 0 |
| SR146132 | 100 | 100 | 100 | 100 | 100 | 99 | 100 | 99 | 99 | 99 | 100 | 100 |
| SR188392 | 100 | 100 | 99 | 99 | 99 | 98 | 99 | 99 | 99 | 99 | 99 | 99 |
| SR188971 | 100 | 100 | 100 | 100 | 100 | 98 | 95 | 100 | 100 | 100 | 100 | 100 |
| SR195750 | 100 | 100 | 100 | 98 | 98 | 100 | 100 | 100 | 100 | 100 | 100 | 98 |
| SR214350 | 100 | 100 | 100 | 100 | 100 | 100 | 100 | 100 | 99 | 99 | 100 | 100 |
| SR237399 | 100 | 100 | 98 | 97 | 97 | 98 | 97 | 87 | 95 | 95 | 98 | 100 |
| SR254481 | 100 | 100 | 99 | 98 | 98 | 99 | 99 | 99 | 98 | 98 | 99 | 100 |
| SR257300 | 100 | 100 | 100 | 100 | 100 | 100 | 97 | 98 | 100 | 100 | 98 | 99 |
| SR263982 | 100 | 100 | 100 | 98 | 95 | 100 | 95 | 98 | 89 | 89 | 100 | 100 |
| SR271250 | 100 | 100 | 74 | 100 | 100 | 74 | 75 | 75 | 75 | 75 | 75 | 75 |
| SR277250 | 100 | 100 | 100 | 52 | 52 | 100 | 99 | 95 | 52 | 52 | 100 | 100 |
| SR307231 | 100 | 100 | 98 | 99 | 99 | 98 | 98 | 98 | 98 | 98 | 98 | 96 |
| SR331095 | 100 | 100 | 98 | 100 | 100 | 90 | 98 | 98 | 98 | 98 | 98 | 100 |
| SR336250 | 100 | 100 | 96 | 98 | 98 | 94 | 95 | 96 | 94 | 94 | 96 | 97 |
| SR358650 | 100 | 100 | 84 | 96 | 96 | 91 | 98 | 96 | 96 | 96 | 94 | 96 |
| SR360800 | 100 | 100 |  |  |  |  |  |  |  |  |  |  |
| SR396160 | 100 | 100 | 99 | 97 | 97 | 98 | 99 | 99 | 99 | 99 | 97 | 97 |
| SR418861 | 100 | 100 | 99 | 100 | 100 | 100 | 99 | 99 | 100 | 100 | 99 | 99 |
| SR452230 | 100 | 100 |  | 11 | 11 | 94 | 97 | 31 |  |  | 93 |  |
| SR520921 | 100 | 100 | 99 | 99 | 99 | 100 | 100 | 100 | 99 | 99 | 100 | 100 |
| SR540686 | 100 | 100 | 98 | 98 | 98 | 100 | 100 | 100 | 100 | 100 | 100 | 100 |
| SR541600 | 100 | 100 | 84 | 84 | 84 | 77 | 77 | 77 | 98 | 98 | 77 | 98 |
| SR559590 | 100 | 100 | 99 | 99 | 99 | 99 | 99 | 99 | 99 | 99 | 95 | 100 |
| SR622408 | 100 | 100 | 99 | 98 | 98 | 98 | 99 | 99 | 98 | 98 | 100 | 99 |
| SR638450 | 100 | 100 | 100 | 87 | 87 | 87 | 98 | 91 | 100 | 100 | 94 | 86 |
| SR641213 | 100 | 100 | 98 | 97 | 97 | 98 | 99 | 99 | 99 | 99 | 99 | 98 |
| SR678133 | 100 | 100 | 98 | 95 | 95 | 98 | 96 | 97 | 97 | 97 | 97 | 98 |
| SR701500 | 100 | 99 | 94 | 99 | 99 | 99 | 99 | 99 | 100 | 100 | 99 | 95 |
| SR721150 | 100 | 100 | 100 | 99 | 99 | 97 | 99 | 97 | 98 | 98 | 97 | 98 |
| SR758876 | 100 | 100 | 99 | 100 | 100 | 100 | 99 | 100 | 100 | 100 | 100 | 100 |
| SR759205 | 100 | 100 | 97 | 99 | 99 | 98 | 98 | 98 | 98 | 98 | 98 | 99 |
| SR800747 | 100 | 100 | 99 | 99 | 99 | 98 | 98 | 98 | 99 | 99 | 99 | 99 |
| SR801894 | 100 | 100 | 100 |  |  | 95 |  |  | 78 | 78 |  | 78 |
| SR843321 | 100 | 100 | 97 | 96 | 96 | 96 | 97 | 97 | 97 | 97 | 97 | 96 |
| SR862600 | 100 | 100 | 100 | 100 | 100 | 99 | 100 | 100 | 100 | 100 | 99 | 100 |
| SR869300 | 100 | 100 | 99 | 99 | 99 | 99 | 99 | 100 | 99 | 99 | 99 | 99 |
| SR914400 | 100 | 99 | 100 | 100 | 100 | 100 | 100 | 100 | 100 | 100 | 99 | 100 |
| SR933600 | 100 | 99 | 99 | 97 | 97 | 99 | 99 | 99 | 98 | 98 | 76 | 99 |
| SR961800 | 100 | 100 | 99 | 100 | 100 | 99 | 98 | 99 | 99 | 99 | 99 | 99 |
| SR969000 | 100 | 100 | 97 | 98 | 98 | 96 | 96 |  | 100 | 100 | 96 | 96 |
| SR1016300 | 100 |  |  |  |  |  |  |  |  |  | 100 |  |
| SR1018400 | 100 |  | 89 | 97 | 99 | 89 | 89 |  |  | 89 | 99 | 99 |
| SR1131900 | 100 | 100 | 100 | 100 | 100 | 100 | 100 | 99 | 99 | 100 | 100 | 100 |
| SR1173300 | 100 | 100 | 74 | 100 | 100 |  | 100 |  |  | 100 | 74 | 100 |
| SR1175500 | 100 |  |  |  |  |  |  |  |  |  |  |  |
| SR1175900 | 100 |  |  |  |  |  |  |  |  |  |  |  |
| SR1201244 | 100 | 100 | 100 | 99 | 99 | 100 | 100 | 100 | 100 | 100 | 100 | 100 |
| SR1207340 | 100 | 100 | 100 | 99 | 99 | 98 | 100 | 100 | 100 | 100 | 100 | 100 |
| SR1251900 | 100 | 100 | 99 | 95 | 95 | 95 | 99 | 95 | 99 | 99 | 97 | 95 |
| SR1291775 | 100 | 99 | 97 | 98 | 98 | 98 | 99 | 99 | 97 | 97 | 99 | 97 |
| SR1355150 | 100 | 100 | 100 | 100 | 100 | 100 | 100 | 100 | 100 | 100 | 94 | 100 |
| SR1358431 | 100 | 100 | 100 | 98 | 100 | 98 | 100 | 100 | 100 | 100 | 100 | 100 |
| SR1385110 | 100 | 100 | 100 | 100 | 100 | 100 | 100 | 95 | 100 | 100 | 100 | 95 |
| SR1532800 | 100 | 100 | 100 | 100 | 100 | 99 | 99 | 100 | 100 | 100 | 99 | 100 |
| SR1568180 | 100 | 100 | 100 | 98 | 98 | 98 | 100 | 100 | 100 | 100 | 100 | 100 |
| SR1587818 | 100 | 100 | 98 | 97 | 97 | 97 | 99 | 98 | 98 | 99 | 98 | 99 |
| SR1604140 | 100 | 95 | 95 | 95 | 95 | 95 | 95 | 95 | 95 | 95 | 95 | 95 |
| SR1605828 | 100 | 99 | 98 | 95 | 95 | 60 | 99 | 95 | 98 | 98 | 93 | 94 |
| SR1678800 | 100 | 100 | 98 | 98 | 98 | 98 | 100 | 99 | 97 | 97 | 100 | 98 |
| SR1678950 | 100 | 100 |  | 96 | 96 |  | 92 | 93 | 95 | 95 |  |  |
| SR1681917 | 100 | 99 |  | 77 | 77 |  | 76 | 88 | 97 | 97 |  |  |
| SR1698200 | 100 | 99 | 99 | 99 | 99 | 99 | 99 | 99 | 99 | 99 | 99 | 99 |
| SR1719800 | 100 | 100 | 97 | 99 | 99 | 97 | 99 | 99 | 98 | 98 | 97 | 96 |
| SR1720792 | 100 | 100 | 100 | 100 | 100 | 100 | 100 | 100 | 100 | 100 | 98 | 100 |
| SR1720816 | 100 | 100 | 100 | 100 | 100 | 100 | 100 | 100 | 100 | 100 | 100 | 100 |
| SR1727893 | 100 | 100 | 93 | 95 | 95 | 95 | 96 | 93 | 96 | 96 | 95 | 98 |
| SR1745900 | 100 | 100 | 97 | 97 | 97 | 98 | 96 | 97 | 93 | 93 | 97 | 97 |
| SR1754950 | 100 | 100 | 100 | 100 | 100 | 100 | 100 | 100 | 100 | 100 | 100 | 100 |
| SR1765900 | 100 | 100 | 100 | 97 | 97 | 98 | 97 | 98 | 97 | 97 | 98 | 99 |
| SR1789300 | 100 | 100 |  | 100 | 100 | 100 | 100 | 100 | 100 | 100 | 100 | 100 |
| SR1806601 | 100 | 99 | 98 | 98 | 98 | 96 | 98 | 98 | 98 | 98 | 98 | 98 |
| SR1808413 | 100 | 100 | 100 | 100 | 100 | 99 | 100 | 100 | 100 | 100 | 100 | 100 |
| SR1811574 | 100 | 100 | 100 | 97 | 97 | 97 | 96 | 96 | 96 | 96 | 97 | 98 |
