# Supplementary material for: A Genome-Wide Analysis of Small Regulatory RNAs in the Human Pathogen Group A Streptococcus
Source: PLoS One. 2009 Nov 2;4(11):e7668. doi: 10.1371/journal.pone.0007668 (PMC2765633; doi:10.1371/journal.pone.0007668)
Supplement: Table S5 — Serotype M1 GAS strains studied. (0.07 MB DOC) [file pone.0007668.s005.doc]

**Table S5**

| **Strain** | **Location** | **Year** | ***emm*** | **Disease or source** |
| --- | --- | --- | --- | --- |
| SF370 | - | 1985 | 1.6 | Wound infection |
| MGAS1508 | Czechoslovakia | 1985 | 1.8 | Scarlet fever |
| MGAS2221 | Australia | 1988 | 1.0 | Scarlet fever |
| MGAS5322 | E Finland | 1989 | 1.0 | Throat |
| MGAS294 | Washington | Late 1980s | 1.0 | Invasive |
| MGAS5087 | Ontario | 1993 | 1.0 | Lower resp. tract |
| MGAS5406 | S Finland | 1994 | 1.23 | Throat |
| MGAS5005 | Ontario | 1996 | 1.0 | Cerebral spinal fluid |
| MGAS9127 | Alberta | Late 1990s | 1.0 | Invasive |
